# Many Changes in Speech through Aging Are Actually a Consequence of Cognitive Changes

**DOI:** 10.3390/ijerph19042137

**Published:** 2022-02-14

**Authors:** Israel Martínez-Nicolás, Thide E. Llorente, Olga Ivanova, Francisco Martínez-Sánchez, Juan J. G. Meilán

**Affiliations:** 1 Facultad de Psicología, Universidad de Salamanca, 37008 Salamanca, Spain; llorentel@usal.es (T.E.L.); meilan@usal.es (J.J.G.M.); 2 Instituto de Neurociencias de Castilla y León, 37007 Salamanca, Spain; olga.ivanova@usal.es; 3 Facultad de Filología, Universidad de Salamanca, 37008 Salamanca, Spain; 4 Facultad de Psicología, Universidad de Murcia, 30100 Murcia, Spain; franms@um.es

**Keywords:** speech analysis, aging voice, cognitive state, MMSE, cognitive impairment

## Abstract

Background: During aging, changes in human speech may arise because of the neurophysiological deterioration associated with age, or as the result of an impairment in the cognitive processes underlying speech production. Some speech parameters show specific alterations under the presence of dementia. The objective of our study is to identify which of these parameters change because of age, cognitive state, or the interaction of both. Methods: The sample includes 400 people over 55 years old, who were divided into four groups, according to their age. The cognitive state of the participants was assessed through the MMSE test and three ranks were stablished. Gender was also considered in the analysis. Results: Certain temporal, fluency, rhythm, amplitude and voice quality parameters were found to be related to the cognitive state, while disturbance parameters changed due to age. Frequency parameters were exclusively influenced by gender. Conclusions: Understanding how speech parameters are specifically affected by age, cognitive state, or the interaction of both, is determinant to advance in the use of speech as a clinical marker for the detection of cognitive impairments.

## 1. Introduction

Aging is generally associated with a persistent cognitive decline that starts around the age of 65 [1,2]. When aging-related changes in cognition do not significantly compromise the cognitive performance and quality of life of the elderly in daily living, it is defined as non-pathological senescence (NPS), or healthy aging. In contrast, when the performance of the elderly in standardized cognitive tests differs significantly from what is expected, according to the age-matching comparative scale, but is not severe enough as to affect their daily living activities, the elderly are said to suffer from mild cognitive impairment (MCI). Finally, Alzheimer’s disease (AD) is said to be developing when cognitive decline is insidious, continuous, and progressive over the years, and seriously affects one or more cognitive domains, such as memory, attention, executive functions or language.

In view of this, the evolution of cognitive domains in older adults may be highly variable, and normative data are needed to relate their performance to a reference aging group [3]. For this assessment, clinicians have routinely used screening tests, with the Mini-Mental State Examination (MMSE), by Folstein, Folstein and McHugh [4], being the most popular, mainly due to its ease of use [5]. Although the MMSE has been used for the screening of AD, it has been more effective in monitoring changes—degree and severity—in the cognitive state. However, it is not without criticism, as some authors underline that it shows a lower sensitivity when monitoring changes in patients with severe dementia [6], or when it comes to differentiating between MCI and the early stages of dementia [7].

In fact, the prodromal, or preclinical, stage of AD is conceptually similar to both MCI and healthy aging in elderly people, who show age-related deficits with no affection of daily life activities. All of them usually display deficits in episodic memory, as well as in attention, reasoning, temporal and spatial perception, and language. As AD progresses, it gives rise to aphasic disorders that involve the impairment of expressive language [8] and lead to a total deterioration of oral communication and, ultimately, mutism [9] in the final stage.

In relation to this impairment in expressive language, it is worth mentioning the difficulties in findings words, since they are a particularly relevant symptom in the early stages of AD. Still, word-finding difficulties are also reported in other groups of older people, both healthy and with MCI, as a consequence of aging [10,11]. A possible explanation for word-finding difficulties in AD could be the dementia-driven disruption in the conceptual, or semantic, knowledge storage, which is known to impair, as a result of neurodegenerative processes [12]. However, many studies suggest that word retrieval difficulties in AD cannot be exclusively attributed to semantic memory deficits [13]. In this respect, some recent research on speakers with AD highlights that disruptions in naming tasks are initially due to lexical retrieval problems and only later to conceptual problems [14]. Word production models for language impairments suggest that semantic, lexical, and phonological stages of lexical retrieval interact and influence each other [15]. In AD, the word retrieval problems could be due to impairments in the frontal executive processes involved in the management of phonological, orthographic, and lexical information of words [16,17], thus, primarily occurring on the phono-lexical level. If this is the case, AD-related deficits in word retrieval could be reflected in phonetic disruptions, such as temporal and prosodic alterations of speech, changes in the ability to control amplitude (shimmer) and frequency (jitter) of voice, as well as in lexical and morphological deviations, like paraphasias. 

The question yet to be answered is whether the changes in expressive language in AD reflect impairments in purely linguistic processes (i.e., in lexical-semantic access), which end up affecting the general cognitive state of a person, whether those changes are the result of general deficiencies in cognitive processing (i.e., executive processes), or whether such changes arise as an interaction between both types of impairments [18]. This question reflects the classical discussion about whether language acts as an independent cognitive system or whether, instead, the cognitive state, including all its underlying processes, is a general information processing tool, of which language is one the cognitive components [19,20]. 

The benefit of specifically examining expressive language and its relationship with the overall cognitive state of a person seems to be supported by several studies that directly relate language impairment to general impairment [21,22,23]. Speech and language impairments in older adults are reported to correlate with the severity of cognitive impairment [24,25]. For instance, aphasic disorders are related to an increased risk of developing dementia, greater severity of deterioration, or its faster progression [26]. 

Given this background, the main objective of this study is to explore the relationship between changes in expressive language associated with age and the degree of cognitive impairment related to these deficits. In our study, we have decided to assess language processes through the lens of speech variables, which make an objective and automatic measure of a non-strictly linguistic nature of the behavioral trait (i.e., speech).

Speech is a complex multistage process that converts conceptual ideas into acoustic signals. Thus, the parameters that compose speech signals are conditioned by both the cognitive processes necessary to produce oral language and the biological characteristics of the speaker, for example, age and gender. As for cognitive conditionality, speech production implies a complex online and parallel processing of different linguistic components, such as the following: formation of a communicative intention; generation, conceptualization and organization of the message; syntactic planning; word retrieval; selection of appropriate morphological forms; sequencing of phonemes; phonetic encoding of the articulatory plans, and articulatory execution [27]. In this sense, there is a very close relationship between the cognitive ability of the speaker and their performance in spoken language. By way of example, speakers with NPS show increased frequency and duration of speech pauses, along with a consequent decrease in speech rate [28]. Such changes are interpreted as the result of difficulties in lexical access [11]. Classical studies [29] established a link between certain temporal parameters of speech that are linked to prosody—such as the distribution of pauses—and the syntactic structure of language production. Recent studies show that pauses in language production occur either in the previous moments or on the limits of syntactic structures to help delimit them [30], appear more frequently before verbs than before names [31], and allow us to interpret the meaning of ambiguous information. Some of these parameters have been proposed as indicators of the speaker’s age, as analyzed from voice. It is the case for speech rate [32], vocal quality parameters, and other variables dependent on spectral analysis, such as the center of gravity and the long-term average spectrum (LTAS). In the case of MCI and AD patients, several speech features have been found to be altered and correlated with performance on several cognitive measures [33]. 

As for the biological featuring, classical speech studies have focused on the clinical characteristics of the aging voice, known as presbyphonia. Presbyphonia refers to those changes in the voice that are due to anatomical and physiological alterations in the vocal tract and the curvature of the larynx, and which cause difficulties in controlling acoustic parameters [34,35]. Usually, it leads to a reduction in the vocal range, a decrease in the fundamental frequency (F0) in female voices (from average levels around 248 Hz to 175 Hz) and an increase in male voices (from 110 Hz to 135–160 Hz), a greater variation in the frequencies (jitter) and amplitude in decibels (shimmer), a reduction in resonance and an increase in speech pauses [36].

In this paper, we aim to explore the relationship between changes in expressive language in older people and their cognitive state through the analysis of speech parameters. Our hypothesis is that those speech parameters that have been shown to be effective biomarkers of AD [37] are directly related to the cognitive state of older adults, as measured by the MMSE. Different studies have addressed the role of speech in relation to the binary classification of dementia vs. non-dementia, but they have not assessed the degree to which speech parameters are related to the severity of cognitive impairment. In our study, we use temporal, amplitude, and frequency features (Appendix A contains all the parameters explored in this study). Moreover, since some parameters are altered by aging, we inquire which of them are linked to biological deterioration, and, therefore, depend on age. In addition, we explore whether the gender variable also influences the evolution of any of these parameters. 

## 2. Materials and Methods

### 2.1. Participants

The sample of this study includes 400 participants over 55 years old, whose data was collected in two different centers. Those participants with NPS or MCI were recruited through the Psychological Attention Service for the Prevention of Cognitive Problems in the Elderly from the University of Salamanca. All of them went through a cognitive assessment with Dem-Detect [38] and were classified either as subjects with NPS (52.3%, MMSE mean = 28.24, sd = 2.13) or subjects with MCI (24%, MMSE mean = 23.63, sd = 4.50) following the criteria of the International Working Group on Mild Cognitive Impairment [39]. The rest of the sample, that is, older adults diagnosed with AD (23.8%, MMSE mean = 20.22, sd = 5.87), underwent the same assessment but were recruited from the State Reference Centre for the Care of People with Alzheimer’s Disease and other Dementias, where they were diagnosed by the Spanish National Health Service following NIA-AA criteria [40].

All participants received education in the European variety of the Spanish language. The baseline enrolment occurred in 2012–2019. The project received the approval of the Ethics Committee of the State Reference Centre for the Care of People with Alzheimer’s Disease and other Dementias of Salamanca (Spain), a center belonging to the Ministry of Social Rights and 2030 Agenda. The study was conducted in accordance with the Declaration of Helsinki and its subsequent amendments, and the European Union regulations concerning medical research. Inclusion criteria of the study involved having signed the informed consent and being Spanish speakers over 55 with at least primary levels of education. Exclusion criteria were a personal history of central nervous diseases, alcohol or substance abuse, or any psychiatric disease, and the presence of severe sensory deficits that could impede the administration of cognitive tests.

The participants were divided into three groups based on their score on the MMSE, which were established by using the cut-off points obtained in the comprehensive review by Arévalo-Rodríguez et al. [41]. The established groups were as follows: the 1st rank, people with less than 23 points (moderate to severe cognitive impairment); the 2nd rank, people between 23 and 27 points (MCI); and the 3rd rank, people over 27 points (NPS). The characteristics of each range are given in Table 1.

Regarding the distribution by gender, a greater number of women than men participated in the study (70.5% vs. 29.5%), but no differences were found between the ranges of the MMSE in their distribution.

In order to explore the effect of age, we categorized participants into the following four intervals: less than 70 years old, between 70 and 79, between 80 and 84, and older than 84. These age ranges were established to obtain homogeneous groups in terms of the number of participants in each of them.

### 2.2. Instruments

Dem-Detect toolkit was used to typify and classify the sample. This assessment battery includes the validated Spanish version [42] of the MMSE [4], which also provides an adjustment of the scores according to the variables of age and educational level (adjusted range 0–32). The sensitivity of the Spanish version of the MMSE is 85.6 and its specificity, 96.1. 

Participants also completed the Goldberg scales for depression and anxiety [43]. Those who obtained a score of >6 on the Goldberg Test, and thus presented severe depression or anxiety, were excluded since recent studies indicate that neuropsychiatric symptoms can influence speech in older adults [44], in particular, depression and anxiety have their own acoustic and prosodic characteristics that may distort the results [45,46].

Audio recordings were made in a sound-proof room with a noise level < 35 dB and a reverberation time of less than one second, using an iPad Air with recording software AURIA (2.31, WaveMachine Labs Inc., Chicago, IL, USA) connected to a microphone (Apogee MiC Plus) placed about 14 cm from the speaker’s mouth. The microphone has a cardioid condenser capsule, a frequency range of 20 Hz–20 kHz and 46 dB of mic preamp gain. 

### 2.3. Procedure

We conducted three sessions of neuropsychological assessment with each participant. The sessions included a complete anamnesis, the assessment of activities of daily living, and a cognitive and psychological evaluation.

The first session included the recording of participants’ speech. The task consisted of reading the first paragraph of “Don Quixote” by Miguel de Cervantes (see Appendix B). The paragraph, in modern Spanish, contains 126 syllables. Importantly, even though the text is not phonetically balanced, it was specifically chosen because the first sentence “En un lugar de la Mancha…” [In a village in La Mancha…] is very familiar to all the participants, while the second one presents a higher semantic and syntactic complexity, which causes strained fluency. The text was displayed on a computer screen in 48-font size to make the reading easier for the participants.

Recordings were made in mono at a sampling rate of 44.1 kHz at 16-bit amplitude quantization. Each recording was analyzed using Praat software (version 6.0). Praat determines pitch using acoustic periodicity detection based on autocorrelation, i.e., the correlating of a time-domain signal with itself [47]. This technique is more accurate, noise-resistant, and robust than alternative methods, such as those based on cepstrum or combs. A pitch floor of 75 Hz and a pitch ceiling of 300 Hz for men and 100–500 Hz for women with a Hanning window length of 0.01 s were used in accordance with the programmers’ recommendations. We have focused on those parameters which proved to be effective in predicting Alzheimer’s disease in previous studies (see Appendix A).

### 2.4. Data Analysis

Statistical analysis was conducted using IBM SPSS Statistics for Windows, Version 26.0. We used an ANOVA with a 2 × 3 × 4 factorial design that included gender, cognitive state, and age. According to our hypothesis, these variables could affect certain speech parameters both during non-pathological and pathological aging. We then broke down the results using post hoc comparisons applying Bonferroni correction.

## 3. Results

An ANOVA test showed age differences between the groups of ranges in cognitive state (F2, 397 = 24.084, *p* < 0.001), with the people in the 1st range < 23 significantly older than those in the 3rd range 23–27 (*p* < 0.001). People in the 2nd range 23–27 were also significantly older than the 3rd range > 27 (*p* < 0.001).

An additional ANOVA test found significant differences between the ranges in terms of years of schooling (F2, 385 = 11.388, *p* < 0.001). Differences occur between the 1st < 23 and the 2nd 23–27 (*p* < 0.05) and 3rd > 27 (*p* = 0.001) ranges of scores on the MMSE.

The results of all the variance analyses for speech parameters are presented in Table 2.

The results break down the analyzed speech variables into the following three different groups: speech parameters that vary depending exclusively on the speaker’s cognitive state; speech parameters that depend exclusively on the speaker’s age, and speech parameters that differ according to gender.

### 3.1. Speech Parameters That Change Depending on the Cognitive State

This group of speech parameters includes variables that showed significant differences between the participants, exclusively due to the main effect of the score range on the MMSE. There were neither interaction effects with age, nor main effects due to age. However, in some cases there were interactions with gender.

Within this first group, temporal parameters, such as total duration time and number of pauses, can be found. Significant differences in total duration time occurred between participants with MMSE < 23 (70 s) and the rest of the categories (MMSE = 23–27, diff: −24 s, *p* < 0.001; and MMSE > 27, diff: −30 s, *p* < 0.001). See Appendix C for a summary table (Table A1) of the post hoc tests in parameters with effects of cognitive state or age.

**Figure 1:**
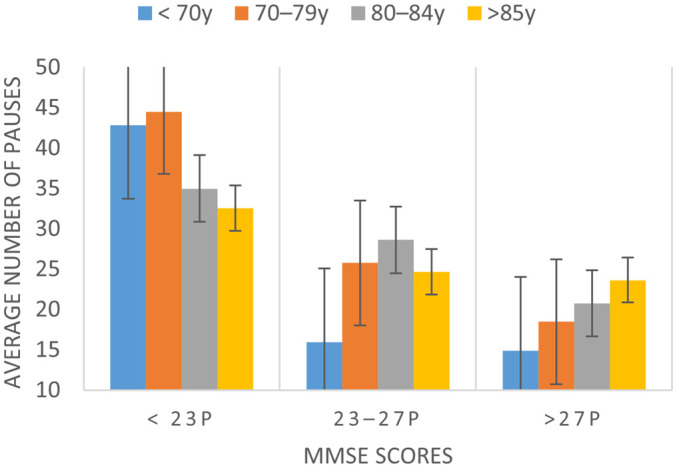
Average number of speech pauses depending on the cognitive state as measured by the MMSE and age ranges.

As for the number of pauses, differences were observed between the participants with MMSE < 23 (39 pauses) and the rest of the categories (MMSE = 23–27, diff: −15, *p* < 0.001; and > 27, diff: −19, *p* < 0.001). Thus, a cognitive state of <23, categorized as cognitive impairment, implies a significant increase in phonation time, as well as in the number of pauses in speech (see Figure 1 as a representative example of a visual representation of this group of parameters).

**Figure 2:**
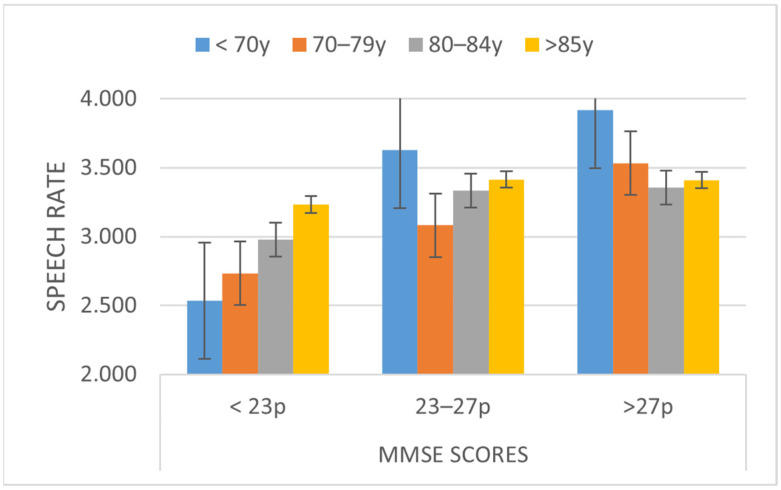
Speech Rate depending on the MMSE and Age Ranges.

The results were similar for the parameters of speech fluency and rhythm, such as speech rate, average duration of syllabic intervals, standard deviation of syllabic intervals and normalized pairwise variability index of syllabic interval (nPVI). In speech rate (phonemes per second), there was an effect in terms of gender, with women (3.42 ph) being faster than men (3.11) (dif = 0.306, *p* < 0.01). However, there was no interaction of gender with either the MMSE or age. There was also an effect of the MMSE that showed differences between the participants with MMSE < 23 (2.87 ph/s) and the rest (MMSE = 23–27, diff: −0.496, *p* < 0.001; and >27, diff: −0.685, *p* < 0.001). Thus, a cognitive state of <23 implies a significant decrease in the speech rate. A significant interaction effect between MMSE and age was found (F6, 376 = 2.385, *p* < 0.05). The differences in the MMSE are present in those who are younger than 70 and MMSE > 27 (See Figure 2). 

We also found changes in several syllabic rhythm parameters. Average duration of syllabic interval showed an effect of cognitive state. Differences were found between participants with MMSE < 23 (0.20 ms) and those with MMSE > 27 (diff: 0.02, *p* < 0.05). Therefore, the average duration of the syllabic intervals is greater in people with moderate cognitive impairment. A significant interaction effect between cognitive state and age was found (F6, 376 = 2.467, *p* < 0.05). The differences between MMSE ranges appear only in those participants under 70 years of age. In this age group, there are differences between MMSE < 23 (0.23 s) and both MMSE 23–27 (diff = 0.04 s, *p* < 0.01) and MMSE > 27 (diff = 0.04 s, *p* < 0.001).

**Figure 3:**
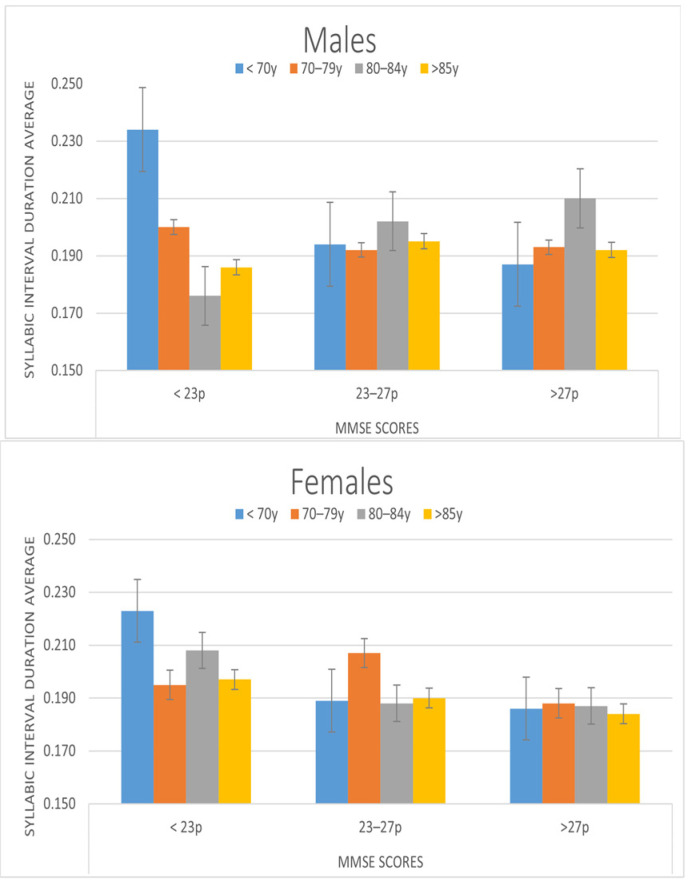
Average Duration of Syllabic Interval depending on the cognitive state as measured by the MMSE and age ranges.

In this parameter we found an interaction effect between cognitive state, age, and gender (F6, 376 = 2.271, *p* < 0.05). In men, there are only differences in the syllabic interval duration (F3, 376 = 2.891) in MMSE < 23, while in women there are differences in MMSE < 23 (F3, 376 = 2.704, *p* < 0.05) and MMSE 23–27 (F3, 376 = 2.683, *p* < 0.05). Figure 3 shows the distribution of the syllabic interval parameters over different ranges of cognitive state for males and females.

Standard deviation of syllabic intervals duration is a parameter that analyzes the average variability of the distances between syllables. Differences were found between participants with MMSE < 23 (0.11 ms) and MMSE > 27 (diff: 0.01 *p* < 0.001). There were also differences between participants with MMSE = 23–27 (0.012) and MMSE > 27 (diff: 0.006 *p* < 0.05). Thus, the rhythm in SD of syllabic intervals shows greater variability when the cognitive state is typical of cognitive impairment.

**Figure 4:**
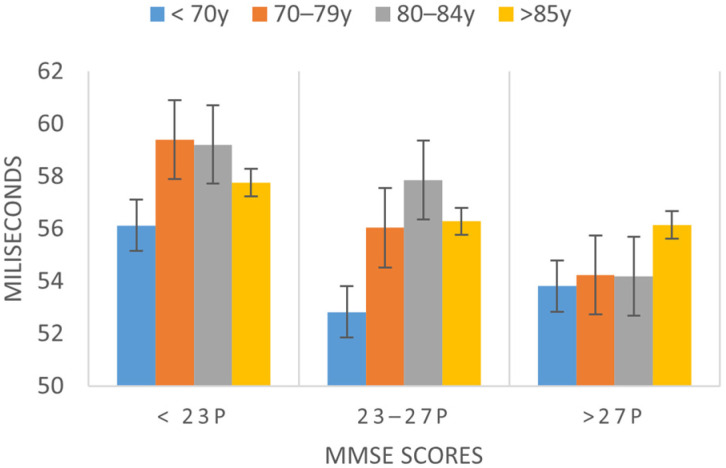
Evolution of the nPVI values depending on the cognitive state as measured by the MMSE and age ranges.

Normalized pairwise variability index (nPVI) is a parameter that analyzes the variability of the distances between syllables. A high nPVI value corresponds to a greater rhythmic variability. In this parameter, differences were observed between participants with MMSE < 23 (58.128 ms) and those with MMSE > 27 (diff: 3.53, *p* < 0.001). This means that the average duration of the syllabic intervals has a significantly more irregular rhythmic pattern when the cognitive state corresponds to cognitive impairment. Figure 4 shows the distribution of nPVI over different ranges of cognitive state. This pattern is similar to that of other syllabic rhythm alteration parameters, such as the average duration of syllabic intervals, and the standard deviation of the average duration of syllabic intervals.

**Figure 5:**
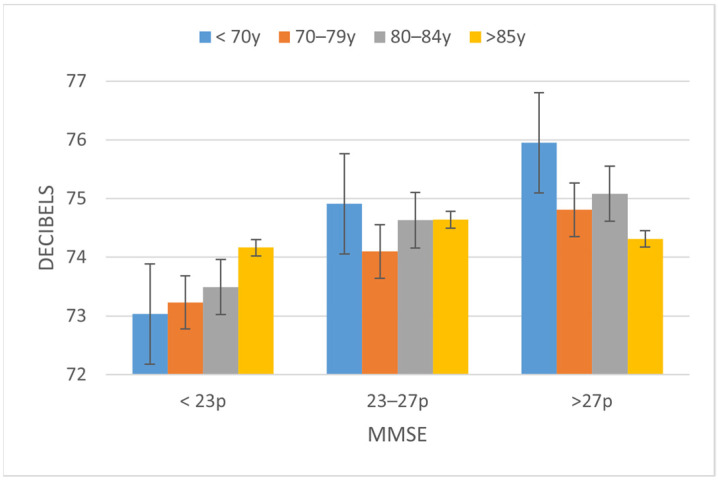
Evolution of the values of the Mean amplitude depending on the cognitive state as measured by the MMSE and age ranges.

Parameters of amplitude and voice quality, such as mean amplitude, long-term average speech spectrum (LTAS), and LTAS 50-1K, also showed significant differences. The mean amplitude parameter reflects the mean energy that the speaker gives to the utterance. Differences were found between participants with MMSE < 23 (73.48 dB) and those in the other categories, MMSE 23–27 (diff: −1.089, *p* < 0.05) and MMSE > 27 (diff: −1.558, *p* < 0.001). Therefore, the average intensity of the voice is lower when the cognitive state corresponds to cognitive impairment. See example in Figure 5.

The LTAS parameter is a voice quality parameter that correlates the in-time adjustment of formant frequencies with their appropriate intensity. This parameter showed significant differences between participants with MMSE < 23 (30.674) and MMSE > 27 (diff: −1.502, *p* < 0.01). Then, the energy of the spectrum is lower when the cognitive state is of cognitive impairment. The LTAS 50-1K parameter is a measure of LTAS that is centered on the spectrum of the first 1000 Hz (from 50 to 1000 Hz). The differences occurred between the participants with MMSE < 23 (43.12 dB) and MMSE > 27 (diff: −1.426, *p* < 0.01). In this sense, the energy of the spectrum in the range of 1000 Hertz is lower when the cognitive state is that of cognitive impairment.

**Figure 6:**
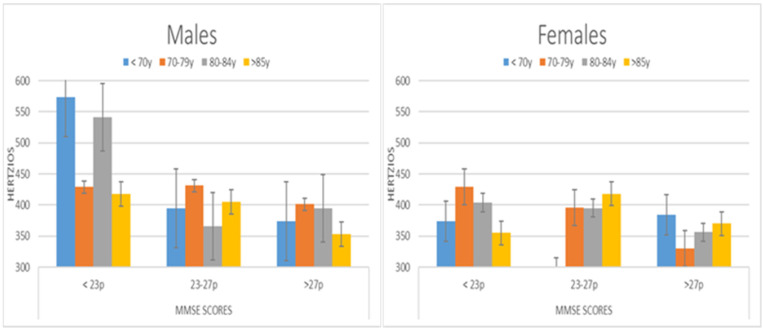
Evolution of the values of the F1sd depending on MMSE and Age Ranges.

Finally, the standard deviation of Formant 1 parameter (F1sd) depends on the cognitive state, but also on gender and age. On the one hand, significant differences were found between participants with MMSE < 23 (440 hz), and both MMSE 23–27 (diff: 54.32 hz; *p* < 0.05) and MMSE > 27 (diff: 69.948 hz, *p* < 0.01). We found no interaction between the two variables of cognitive state and age. However, we found differences due to gender between men (423 hz) and women (374 hz, *p* < 0.01). In contrast, we did find interaction when the following three variables are compared: age, gender and cognitive state. The differences were found in women with a cognitive state between 23 and 27 points in the MMSE, and under 70 years of age with respect to other groups. There were also differences between men and women younger than 79 years of age and MMSE < 27 (see Figure 6).

### 3.2. Parameters That Change Depending on Age

This section analyzes those speech parameters that showed significant differences between participants due to the main effect of age, i.e., harmonics to noise ratio (HNR) and jitter (local). There were no interaction effects of age with cognitive state and gender, although in both cases, a main effect from gender was found. 

Harmonics to noise ratio (HNR) measures the proportion of energy of the harmonic components of the voice (periodic sound) to noise within it (aperiodic). There were differences due to gender, the amplitude of the harmonics being lower in males (10.988 dB vs. 12.994 dB). There were differences due to age, being the amplitude of the harmonics lower in >85 old than in <70 old (11.274 dB vs. 12.982 dB, respectively). We did not find any interaction effect between age and gender variables.

Jitter (local) measures the percentage of average frequency variation between two consecutive periods. Again, we found differences by gender, the variability being greater in males (2.936% vs. 2.400%). There were differences due to age, the jitter loc being lower (*p* < 0.05) in >85 old than in <70 old (2.340% vs. 2.834%, respectively). However, we did not find interaction effect. Thus, the older the people, the greater the percentage of variability in the frequencies. 

### 3.3. Parameters That Change Depending on Gender

These speech parameters showed no main effects of MMSE or age; we did not find interaction effects between the variables either. These parameters are frequency based and rely only on the gender of the speaker and are, therefore, closely related to biological aspects. This is the case of the F0 mean, since older men (138 Hz) maintain a lower mean tone than women (180 Hz). This is also true of spectral skewness, where the center of gravity of the mean frequency is more positive towards low frequencies in men (10.199 Hz) than in women (8.389 Hz).

## 4. Discussion

In this study, we have explored a wide range of speech parameters and the following three factors that may determine their change in older adults: cognitive state, age, and gender. Many of the parameters seem to be determined by the speaker’s cognitive state, as measured by the MMSE. Therefore, age-related neurodegenerative evolution towards dementia will directly affect the speech production processes related to these variables. Speech does not depend on a single process, but rather requires a complex of neurocognitive multistage processes for its execution. Any age-related alteration in such processes will, consequently, affect speech properties and production. Considering this, in this study, we have analyzed which speech variables are cognitively driven and are, thus, determined by the cognitive state of the older adult, and which of them depend more on biological factors, such as age or gender. Understanding how older adults produce speech and how its parameters can be affected by cognitive state, age, and gender, is highly relevant, both for monitoring speech changes throughout the aging process, and for supporting their use as clinical markers for the diagnosis of neurodegenerative diseases.

In the present research, we have identified several speech parameters, whose expression varies in accordance with the scores obtained by the elderly on the MMSE. Thus, the greater the cognitive impairment in older adults, as measured by this test, the longer the phonation time, the more pauses in speech, the lower the rate of speech, the greater the mean duration of syllabic intervals, and the greater variability in the syllabic intervals. Altogether, these alterations would reflect difficulties in lexical retrieval and a specific impairment of cognitive control. The nature of the speech parameters we have identified, as subject to the cognitive state condition, confirms this assumption. 

During speech production, empty pauses are more frequent and longer near to the syntactic limits of the sentences or when sentences based on low-frequency words are uttered [48]. Thus, speech output is conditioned by the pressure of having to syntactically plan sentences while producing two or three syllables per second. 

Another factor that may determine the importance of the syllabic aspects in age-related speech production is the fact that Spanish is a syllable-timed language [49], which means that every syllable takes approximately the same amount of time to be pronounced, regardless of being stressed or not. Therefore, the rhythm in the Spanish language is monotonous, and non-pathological speech is defined by the regularity in the syllabic intervals. As we have shown, such regularity is altered in moderate cognitive impairment.

Similarly, intensity and voice quality parameters, as mean amplitude, LTAS and LTAS 50-1K, also appear to depend on the cognitive state of the older adult. We have found a significant decrease in mean intensity, associated with cognitive deterioration, and we suggest that this could be a feasible explanation to an age-related decrease in amplitude, observed in other studies [50]. Even though there is enough evidence supporting a weakening of the voice with age, little is known about the reason for this [51]. It has been proposed that the decrease in intensity could be due to a compensatory muscular and phonatory effort [52]. Regarding LTAS, for its part, this correlates with the vocal effort [53] involved in the laryngeal and phonetic-phonological adjustments that occur in the supraglottic cavities, and it is significantly related to the severity of dysphonia [54]. In this way, higher values in this parameter are related to a harsh, hoarse voice, with a poverty of harmonic elements at high frequencies; low values, associated with dementia, show low vocal effort with harmonic inadequacy at low frequencies. 

Similarities in the evolution of these parameters, depending on the cognitive state across groups, are noteworthy. Total duration, pauses, speech rate, amplitude and F1 do not show differences between 23–27 and <27 ranges. The nPVI, LTAS, and LTAS 50-1K parameters only show differences between <23 and <27. These parameters evolve slowly and reveal very slight changes, before becoming significant only at moderate cognitive impairment stage. However, average duration of syllabic interval and standard deviation of syllabic intervals duration did differentiate between older people with MCI and those with NPS.

Therefore, the global cognitive state of the older adults seems to be closely related to the production of connected speech, which stands as a useful behavioral measure for processing ability. Difficulties in accessing lexicon and syllabic structures, driven by general cognitive impairments, would slow down the speech rate and affect rhythm and prosody [11,28]. Thus, identifying through speech the specific processes that change at the early stages of dementia will be important for developing more effective diagnostic procedures.

The speech parameters modulated by age as a biological factor are vocal disturbance features, such as HNR and jitter loc, related to the loss of speech quality [50]. According to the literature, confirmed in our results, HNR is lower in men and decreases with age, causing the typical harness of the aging voice. In both men and women, we have found a HNR of less than 20 dB, which depicts an impaired value. Jitter loc was greater in men than in women, and, in line with other studies [55], we have observed that this increases with age. Such disturbances are usually attributed to deficits in the neurological control of the muscles, as well as to mechanical changes due to atrophy of the vocal cords. 

Finally, some of the speech parameters proved to be fundamental in the prediction of AD by other studies [56,57], have been shown to solely depend on gender—neither on age nor on cognitive state—in the present research. This is the case for frequency and spectral analysis parameters, such as F0 and asymmetry. In this regard, we have found that men showed a lower tone and a more pronounced tendency towards low frequencies than women. This result does not coincide with most studies [35,55], which maintain that there is an age-related decrease in F0 in women while it would increase in men. F0 is commonly considered to be determined by gender and age because of its direct dependency on the mass and length of the vocal folds. However, we have found no significant changes in this respect. Regarding spectral skewness, this is also determined by the physical deterioration of ligaments and cartilage, and by a more pronounced curvature of the larynx at the margins, causing a change in the frequency parameters after age 55. Nevertheless, our data have not shown any changes in this parameter due to aging, neither in men nor in women.

## 5. Conclusions

We have verified that temporal, amplitude, and voice quality parameters may serve as an objective measure of language cognitive processes. We suggest that these parameters measure executive processes involved in language production. In this respect, our results support those theoretical models of aging that defend that there is a reduction in processing resources, both in successful and pathological aging. Attentional resources supporting cognitive processing decline with age, which leads to deficits in prefrontal executive control processes [58,59]. By comparison, voice disturbance parameters seem to be related to non-cognitive motor articulatory processes. We have not found any interaction effect between them; while the first group of speech parameters depends on the general cognitive state of the elderly, the second group of speech variables does so on the mere passage of age, and they independently influence expressive language production. 

The main novelty of this study is that we go beyond the classical binary classification of groups as dementia vs. non-dementia. This has allowed us to identify a set of speech parameters that are able to quantify the severity of cognitive impairment, as measured by the MMSE. With this, we confirm our initial hypothesis, and we suggest that our results open the door to explaining the relationship between speech parameters and their underlying cognitive processes.

Even so, some results from the present study are not consistent enough. This is the case with the role of spectral analysis parameters in identifying the degree of cognitive impairment. The literature suggests that no phonological changes occur in aging due to cognitive impairment; however, in this study, altered spectrographic parameters were found, related to the early stages of cognitive impairment [60]. We suggest that such changes could depend on other factors that have not been addressed in this study. 

One downside of brief cognitive assessment tools is that they can result in a misclassification of dementia compared to a gold-standard diagnosis [61,62]. Therefore, it is important to consider age, gender and education when interpreting the MMSE scores. For instance, a low educational level and socioeconomic status are associated with lower MMSE scores [63]. These are some of the limitations of the MMSE test, regarding its effectiveness in detecting cognitive impairment. 

This is also a limitation in our study. We have found differences in schooling between the groups, and which primarily affects the group with higher cognitive impairment. This group has fewer years of schooling and is also significantly older than the other two groups. Although it seems that age does not play a relevant role in the parameters related to the MMSE, schooling remains a factor to be explored in the future. 

These variables and some others, such as encoding or learning capacity, could contribute to the ability to express speech, and it would be necessary to explore their role to a greater extent. We hope that the exploration of the speech parameters and of the different factors affecting their performance will contribute to explaining and optimizing the screening tools based on the automatic speech analysis.

## Figures and Tables

**Table 1:** Characteristics of the sample divided by the MMSE scoring.

	N	AGE Mean (sd)	Schooling Years Mean (sd)	MMSE Mean (sd)
1st Range MMSE < 23*p*	97	81.37 (8.60)	7.66 (3.59)	17.68 (4.37)
2nd Range MMSE 23–27*p*	115	78.80 (8.04)	9.00 (3.98)	25.23 (1.44)
3rd Range MMSE > 27*p*	188	74.29 (8.93)	10.03 (4.05)	29.12 (0.96)
Total	400	77.31 (9.09)	9.16 (4.03)	25.23 (5.16)
Men	118	76.60 (9.31)		
Women	282	77.60 (8.99)		

**Table 2:** Influence of gender, cognitive state (MMSE), and age categories on the speech parameters involved in the prediction of AD.

Parameters	Gender F_(1, 376)_	Interaction MMSE-Age F_(6, 376)_	MMSE F_(2, 376)_	Age F_(3, 376)_
Duration (oral reading time)	0.142	0.620	28.140 ***	1.244
Number of Pauses	0.153	0.439	28.477 ***	0.959
Speech Rate	10.845 *	0.353	16.835 ***	1.725
Average duration of syllabic intervals	0.254	2.271 *	3.886 *	1.646
Standard Deviation of syllabic intervals duration	2.058	0.936	10.917 ***	0.315
nPVI	0.622	0.356	6.408 ***	1.973
Mean Amplitude	1.759	0.560	8.202 **	0.624
LTAS	0.005	0.581	5.103 **	2.616
LTAS_50-1K	1.638	0.869	6.217 **	1.134
F1 sd	11.507 **	2.130 *	7.253 **	0.562
F0	170.203 ***	0.689	2.654	0.992
Spectral Skewness	5.649 *	1.116	0.932	0.582
HNR	27.830 ***	1.011	1.137	2.877 *
Jitter (Local)	27.740 ***	0.968	0.805	3.427 *

* *p* < 0.05; ** *p* < 0.01; *** *p* < 0.001.

## Data Availability

The data that support the findings of this study are available from the corresponding author upon reasonable request.

## Appendix A

Speech parameters analyzed in this study.

(1) Total duration (seconds) of phonation time (oral reading time) with sufficient sound quality.
(2) Number of pauses: short interruptions of more than 250 ms in duration. Even though little is known about how they are planned, we do know that they occur in the previous moments or in the limits of syntactic structures to help delimit them, appear more frequently before verbs than before names, and allow us to interpret the meaning of ambiguous information.
(3) Speech rate: total number of phonemes produced, divided by the total duration of the utterance.
(4) Average duration of syllabic intervals: speech rhythm can be identified in the acoustic signal and on a perceptual level as the repetition of a regular pattern of a prosodic constituent. Rhythm depends on syllabic structure, phonetic vowel reduction and the position of the stress. The average duration of the syllabic interval looks for a pattern of regular distances between syllables. Syllable-timed languages are characterized by maintaining this regularity.
(5) Standard deviation of syllabic intervals duration: mean standard deviation of the duration of the syllabic intervals.
(6) Normalized pairwise variability index (nPVI): the normalized index of variability by pairs. It is a normalized measure of the speech rate variability in syllable durations. It is calculated by the mean of the differences in duration between two successive speech intervals (Vs), divided by the sum of those intervals. It is a refined index, as it measures the percentage difference in rhythm between adjacent intervals, instead of a total mean. Each vowel pair is normalized in relation to the arithmetic mean of that pair. A high nPVI value corresponds to higher rhythmic variability, characteristic of stress-timed languages, whereas low nPVI values are typical of syllable-timed languages, in which the syllables take approximately equal amounts of time to be pronounced.
(7) Mean amplitude: average of intensity values in an utterance. The standard is usually 60.05 dB.
(8) Long-term average speech spectrum (LTAS): average of the several successive spectra of the signal eliminating the silence segments. It is used to analyze voice quality, i.e., speaker’s phonetic-phonological adjustments. This is reflected in the quality of the frequency peaks when adjusting the appropriate frequencies at each moment (Hz) or the appropriate energy or intensity to each formant (dB).
(9) LTAS_50-1K: result of analyzing high frequency energy in the range of 50 to 1000 Hz using the long-term average spectrum (LTAS).
(10) Fundamental frequency (F0 mean): average pitch in an utterance or voice period that corresponds to the number of times vocal cords open and close per second. Its values are 208.25 Hz for women, 121.86 Hz for men; on average, 176.42 Hz.
(11) The formant F1 is a concentration of acoustic energy around 500 Hz in the speech wave. The F1 sd is standard deviation mean of F1.
(12) Spectral skewness: it indicates whether the center of gravity of the average frequency is skewed to high frequencies (negative asymmetry), to low ones (positive asymmetry), or in in the center (medium frequencies, symmetric distribution).
(13) Harmonics to noise ratio (HNR): measure, in decibels, of the periodic harmonic energy produced by vocal folds vibration, with respect to the aperiodic additive noise (non-harmonic energy) that can be found in the voice signal. Therefore, it assesses the harmonicity or degree of acoustic periodicity and, the smaller it is, the more noise present and the greater the degree of dysphonia.
(14) Jitter (local): mean of the pitch variation made period by period. The normality threshold is 1.04%, and it is calculated by dividing the absolute average difference of the frequency between consecutive periods by the total average frequency of the signal periods (average period).

## Appendix B

### Appendix B.1. Translated Version

In a village of La Mancha, the name of which I have no desire to call to mind, there lived not long since one of those gentlemen that keep a lance in the lance-rack, an old buckler, a lean hack, and a greyhound for coursing. An olla of rather more beef than mutton, a salad on most nights, scraps on Saturdays, lentils on Fridays, and a pigeon or so extra on Sundays, made away with three-quarters of his income.

### Appendix B.2. Original Version

En un lugar de la Mancha, de cuyo nombre no quiero acordarme, no ha mucho tiempo que vivía un hidalgo de los de lanza en astillero, adarga antigua, rocín flaco y galgo corredor. Una olla de algo más vaca que carnero, salpicón las más noches, duelos y quebrantos los sábados, lantejas los viernes, algún palomino de añadidura los domingos, consumían las tres partes de su hacienda.

## Appendix C

**Table A1:** Mean difference in pairwise comparisons of parameters with a main effect of age or cognitive state. * is significant at *p* < 0.05 and ** is significant at *p* < 0.001.

Pairwise Comparisons	Duration	Number of Pauses	Speech Rate	Average Duration Syllabic Intervals	sd of Syllabic Intervals Duration	nPVI	Mean Amplitude	LTAS	LTAS 50-1k	F1 sd	HNR	Jitter (Local)
<70 vs. 70–79	−7.997	−5.032	0.245	0.006	0.001	−2.301	0.587	0.551	0.815	−5.576	0.974	−0.407
<70 vs. 80–84	−9.087	−3.567	0.138	0.007	−0.001	−2.827	0.229	−0.542	0.266	−12.4	1.279	−0.41
<70 vs. >85	−6.883	−2.407	0.008	0.011	0.000	−2.472	0.26	−0.578	0.487	10.858	1.711 *	−0.493 *
70–79 vs. 80–84	−1.09	1.465	−0.106	0.001	−0.003	−0.527	−0.358	−1.093	−0.549	−6.824	0.305	−0.002
70–79 vs. >85	1.114	2.625	−0.237	0.005	−0.001	−0.171	−0.326	−1.129	−0.328	16.434	0.736	−0.086
80–84 vs. >85	2.204	1.160	−0.131	0.005	0.001	0.355	0.032	−0.036	0.221	23.258	0.431	−0.083
<23 vs. 23–27	23.590 **	14.876 **	−0.496 **	0.008	0.007	2.374	−1.089 *	−1.156	−0.979	54.372 *	0.371	0.042
<23 vs. >27	29.988 **	19.28 **	−0.685 **	0.012 *	0.013 **	3.533 *	−1.558 **	−1.502*	−1.426 *	69.948 *	0.704	−0.088
23–27 vs. >27	6.398	4.304	−0.189	0.004	0.006 *	1.16	−0.469	−0.347	−0.448		0.333	−0.13
